# Informing randomized clinical trials of respiratory syncytial virus vaccination during pregnancy to prevent recurrent childhood wheezing: A sample size analysis

**DOI:** 10.1016/j.vaccine.2018.10.041

**Published:** 2018-12-18

**Authors:** Corinne A. Riddell, Niranjan Bhat, Louis J. Bont, William D. Dupont, Daniel R. Feikin, Deshayne B. Fell, Tebeb Gebretsadik, Tina V. Hartert, Jennifer A. Hutcheon, Ruth A. Karron, Harish Nair, Robert C. Reiner, Ting Shi, Peter D. Sly, Renato T. Stein, Pingsheng Wu, Heather J. Zar, Justin R. Ortiz, for the WHO Technical Working Group on Respiratory Syncytial Virus Vaccination During Pregnancy to Prevent Recurrent Childhood Wheezing

**Affiliations:** a Division of Epidemiology & Biostatistics, University of California, Berkeley, 2121 Berkeley Way, Suite 5404, Berkeley, CA, USA; b Center for Vaccine Innovation and Access, PATH, 2201 Westlake Ave, Seattle, WA, USA; c Department of Paediatrics, Wilhelmina Children’s Hospital, University Medical Centre Utrecht, Lundlaan 6, Utrecht, the Netherlands; d The ReSViNET Foundation, Zeist, the Netherlands; e Department of Biostatistics, Vanderbilt University School of Medicine, Suite 1100, Room 11119, 2525 West End Ave., Nashville, TN 37203-1741, USA; f Initiative for Vaccine Research, World Health Organization, 20 Avenue Appia, Geneva, Switzerland; g School of Epidemiology and Public Health, University of Ottawa, Children’s Hospital of Eastern Ontario (CHEO) Research Institute, 401 Smyth Road, CPCR, Room L-1154, Ottawa, Ontario K1H 8L1, Canada; h Center for Asthma Research, Vanderbilt University School of Medicine, Department of Biostatistics, 2525 West End Ave, Suite 11000, Nashville, TN 37203, USA; i Center for Asthma Research, Allergy, Pulmonary & Critical Care Medicine, Vanderbilt University School of Medicine, 2525 West End Ave, Suite 450, Nashville, TN 37203, USA; j Department of Obstetrics & Gynaecology, University of British Columbia, Shaughnessy C408A, British Columbia Children’s & Women's Hospital, 4500 Oak Street, Vancouver, British Columbia V6H 3N1, Canada; k Center for Immunization Research, Johns Hopkins University, 624 N. Broadway, Suite 217, Baltimore, MD, 21205, USA; l Centre for Global Health Research, Usher Institute of Population Health Sciences and Informatics, University of Edinburgh, Medical School, Teviot Place, Edinburgh EH8 9AG, Scotland, United Kingdom; m Department of Global Health, University of Washington, 2301 5th Ave, Suite 600, Seattle, WA 98102, USA; n Child Health Research Centre, University of Queensland, 62 Graham St., South Brisbane, QLD 4101, Australia; o Pediatric Pulmonary Unit, Pontificia Univeridade Católica RS, Av. Ipiranga, 6690/420 Porto Alegre, Brazil; p Division of Allergy, Pulmonary, and Critical Care Medicine, Vanderbilt University Medical Center, 2525 West End Ave, Suite 1130, Nashville, TN, USA; q Department of Paediatrics and Child Health, Red Cross War Memorial Children's Hospital, Cape Town, South Africa; r SA-Medical Research Council Unit on Child and Adolescent Health, University of Cape Town, 5th Floor ICH Building, Klipfontein Road, Cape Town, South Africa; s Center for Vaccine Development, University of Maryland School of Medicine, 685 W. Baltimore St, Suite 480, Baltimore, MD, USA

**Keywords:** Respiratory syncytial virus, Vaccine, Wheeze, Asthma, Global health, Pregnant

## Abstract

**Background:**

Early RSV illness is associated with wheeze-associated disorders in childhood. Candidate respiratory syncytial virus (RSV) vaccines may prevent acute RSV illness in infants. We investigated the feasibility of maternal RSV vaccine trials to demonstrate reductions in recurrent childhood wheezing in general paediatric populations.

**Methods:**

We calculated vaccine trial effect sizes that depended on vaccine efficacy, allocation ratio, rate of early severe RSV illness, risk of recurrent wheezing at age 3, and increased risk of RSV infection on recurrent wheezing. Model inputs came from systematic reviews and meta-analyses. For each combination of inputs, we estimated the sample size required to detect the effect of vaccination on recurrent wheezing.

**Results:**

There were 81 scenarios with 1:1 allocation ratio. Risk ratios between vaccination and recurrent wheezing ranged from 0.9 to 1.0 for 70% of the scenarios. Among the 57 more plausible scenarios, the lowest sample size required to detect significant reductions in recurrent wheezing was 6196 mother-infant pairs per trial arm; however, 75% and 47% of plausible scenarios required >31,060 and >100,000 mother-infant pairs per trial arm, respectively. Studies with asthma endpoints at age 5 will likely need to be larger.

**Discussion:**

Clinical efficacy trials of candidate maternal RSV vaccines undertaken for licensure are unlikely to demonstrate an effect on recurrent wheezing illness due to the large sample sizes likely needed to demonstrate a significant effect. Further efforts are needed to plan for alternative study designs to estimate the impact of maternal RSV vaccine programs on recurrent childhood wheezing in general populations.

## Highlights

• Candidate RSV vaccines are under development.
• Early infant RSV illness is associated with subsequent wheeze-associated disorders.
• We estimated sample sizes needed for vaccine trials to prevent these outcomes.
• RSV vaccine trials will likely not be large enough to detect reductions in wheeze.
• Alternative study designs to demonstrate RSV vaccine impact on wheeze are needed.

## 1 Introduction

The development of safe and effective vaccines to protect young children against respiratory syncytial virus (RSV) illness is a global health priority [1], [2]. RSV is a major cause of lower respiratory infections (LRI) among young children globally [3], [4]. While nearly all children will have been infected by RSV by two years of age [5], the infections during the first months of life can be the most severe [6]. In addition to its acute effects, early childhood RSV illness has been associated with subsequent development of wheeze-associated disorders later in life [7], [8]. Vaccination of pregnant women against RSV may protect their infants against RSV illness during their first months of life, primarily through maternal antibodies transported across the placenta [1]. While licensure of RSV vaccines for use during pregnancy is likely to be sought for the primary indication of preventing acute RSV illness in young infants, the public health value of maternal RSV vaccines would be greater if the vaccine also prevented wheeze-associated disorders [1]. Studies to assess the effects of RSV prevention on these childhood respiratory outcomes have been recommended by experts convened by the Bill & Melinda Gates Foundation [7], the Lancet [9], and the World Health Organization (WHO) [2]. However, the sample sizes required to detect an effect on such outcomes in randomized clinical trials (RCTs) have not been calculated.

Wheeze-associated disorders in childhood are a common cause of morbidity globally. Wheezing can be severe and can lead to decreased quality of life, frequent healthcare utilization, and high economic costs in young children [9], [10], [11], [12]. Recurrent wheezing may resolve as children age, but for others, the symptoms can persist [10], [12]. The diagnosis of asthma as a cause of recurrent wheezing cannot be objectively made using lung function until about 5 years of age [7], [10]. Definitions for recurrent childhood wheezing typically include multiple episodes of wheezing since birth among children younger than 5 years of age [8], [13], [14].

In 2017, the WHO Initiative for Vaccine Research assembled a technical working group to estimate the sample size required for RCTs of RSV vaccination during pregnancy to demonstrate an effect on recurrent childhood wheezing. This report describes the output of the deliberations. The goal of the work was to inform public health expectations and planning for maternal RSV vaccines, so the working group focused on studies in general communities, rather than in high-risk subgroups.

## 2 Methods

### 2.1 Study design

We estimated the RCT sample size that would be required to demonstrate an effect of RSV vaccination during pregnancy on recurrent childhood wheezing through 3 years of age. We chose this endpoint because recurrent childhood wheezing is an important cause of paediatric morbidity [10], [12]. Additionally, RCTs with recurrent childhood wheezing as a primary endpoint will likely be more favourable than other childhood respiratory outcomes for several reasons. First, an outcome that could be assessed at a younger age is advantageous, because longer trials have higher rates of losses to follow-up and are more costly. Second, recurrent wheezing at 3 years is expected to have a higher prevalence than asthma at 5 years [12], [15], increasing the statistical power for a given sample size. Third, if some portion of asthma at 5 years is caused by early infant RSV exposure, it is plausible that recurrent wheezing at 3 years mediates this relationship. Fourth, atopy and environmental risk factors are more likely to be found in children with asthma than in children with recurrent wheeze [9], [14], [16], [17], indicating that early RSV illness may contribute a larger attributable fraction to recurrent childhood wheezing at 3 years than to asthma at 5 years.

We based our approach on an earlier study that estimated the detectable risks in observational studies of potential foetal benefits of maternal influenza vaccination [18]. The approach is described in the following illustrative example. Suppose that 1000 mother-infant pairs were randomized to each arm of a placebo-controlled RCT of a maternal RSV vaccine. If the baseline risk of vaccine-preventable early infant RSV illness in the population is *r*, then approximately 1000 × *r* infants born to women in the placebo arm will acquire RSV illness during the vaccine-preventable period. In the vaccine arm, prevention of infant RSV illness is proportional to the vaccine efficacy (%), *VE*, implying that 1000 × *r* × (1 − *VE*) infants born to women immunized against RSV will acquire an RSV illness. Supposing an attack rate of 20% (*r*) and vaccine efficacy of 50% (*VE*), we would expect 200 cases (= 1000 × 0.2) of RSV illness among infants in the placebo arm and 100 cases (=1000 × 0.2 × 0.5) of RSV illness among infants in the vaccine arm.

**Fig. 1:**
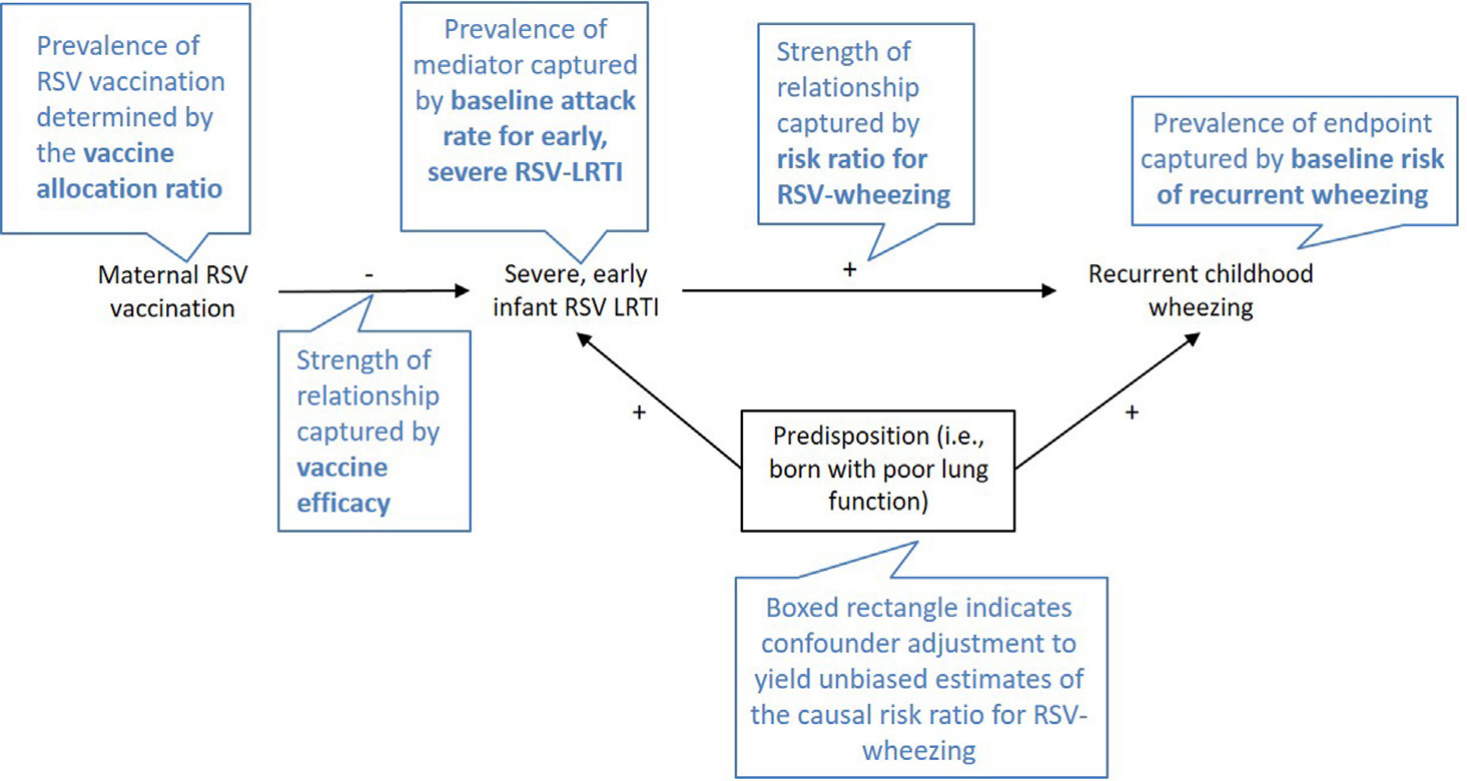
Theoretical causal diagram for the relationships between maternal RSV vaccination, severe early infant RSV- lower respiratory infections, and later recurrent childhood wheezing. The diagram illustrates how maternal vaccination against RSV may prevent the development of recurrent childhood wheezing (the endpoint) through preventing an early severe RSV-related lower respiratory infection (LRI) during infancy (the mediator). Links between elements in the diagram and parameters in the sample size study are described in the blue caption boxes. The sign labelling each arrow indicates the direction of association as positive (+) or negative (−) between the connecting nodes. If the relationship between early severe RSV-LRI and recurrent childhood wheezing is confounded by a predisposition to respiratory infections, then observational studies estimating the increased risk of recurrent childhood wheezing due to early RSV-LRI may be overestimated.

**Fig. 2:**
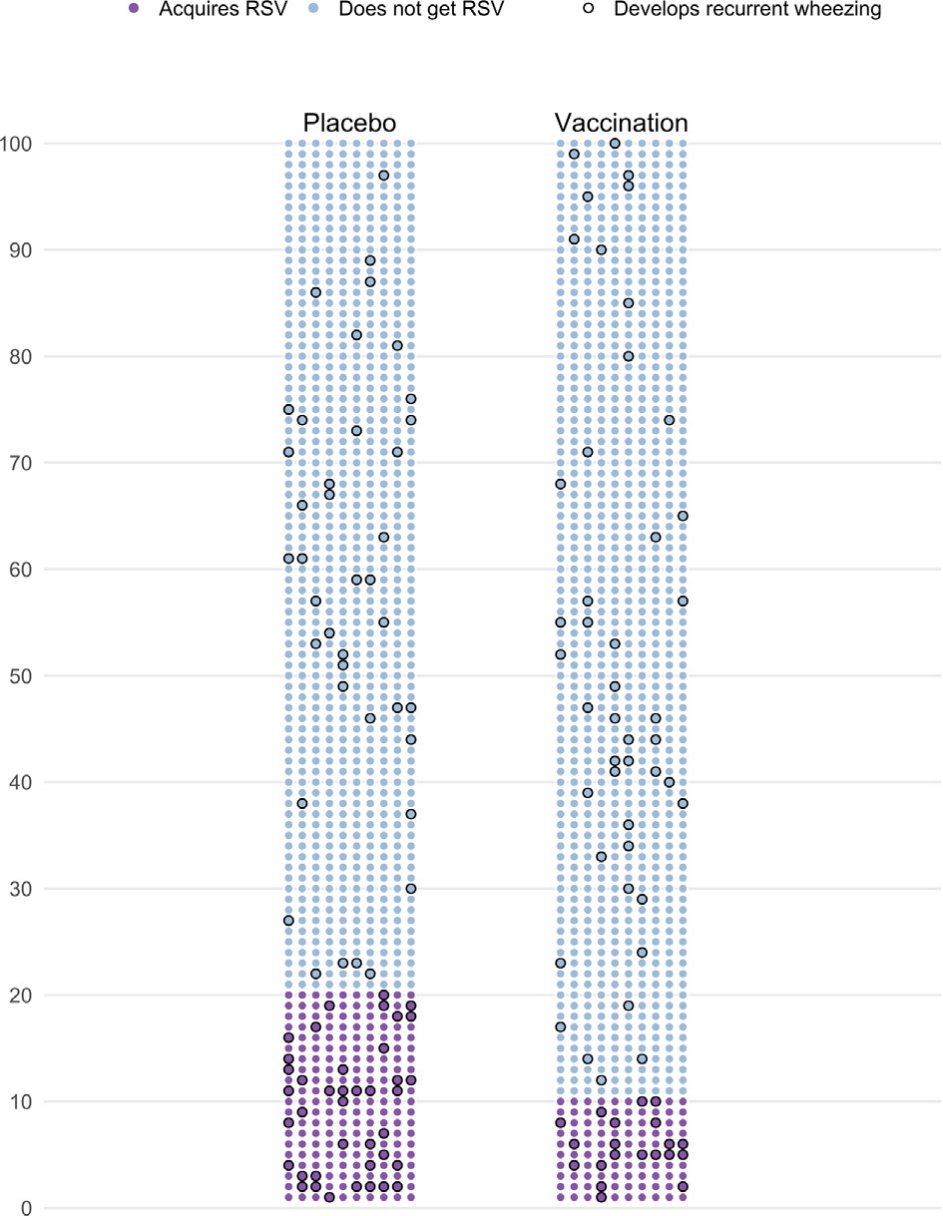
Illustration of the parameters used to estimate risk ratios and sample size in clinical trials of maternal RSV immunization on development of later recurrent childhood wheezing. Each filled dot in this figure represents a mother-infant pair, with the colour representing their RSV infection status and black outline indicating infants who go on to develop recurrent childhood wheezing. There are 100 rows of 10 dots, to represent 1000 mother-infant pairs randomized each to placebo and vaccination. Following the in-text example, 20% of placebo (200 mother-infant pairs; purple dots) acquire early and severe infant RSV vs. 10% in the immunized arm (100 mother infant pairs; purple dots), for a vaccine efficacy of 50%. If early RSV illness increases the later development of recurrent childhood wheezing, then the proportion of children who develop recurrent wheezing will be higher among those with early RSV. This is shown by the higher density of recurrent wheezing cases (black outline) among those with RSV illness (purple dots) vs. those without (blue dots). Summing the wheezing cases, there are 60 cases among the 300 infants who acquired RSV (for a 20% risk) vs. 85 wheezing cases among the 1700 infants who did not acquire RSV (for a 5% risk) giving rise to four-fold increased risk of wheezing in children exposed vs. unexposed to early and severe infant RSV. This example shows 80 cases of childhood recurrent wheezing among the placebo arm vs. 65 among the immunized arm for a risk ratio between vaccination and childhood recurrent wheezing (RR_VW_) of 0·81.

We considered infant RSV illness within the first months to be a mediator on the causal pathway of maternal RSV vaccine protection against recurrent childhood wheezing since these early RSV illnesses occur within the vaccine-preventable time frame and are associated with the highest acute morbidity (Fig. 1). That is, the ability of RSV vaccination during pregnancy to prevent later recurrent childhood wheezing would operate through the reduction in early RSV illness during the first months of life. If the baseline risk of recurrent wheezing at 3 years, *w*, is 5% among infants who do not experience an early RSV illness, and 20% among those who do, this would imply a 4-fold relative increase in the risk resulting in a risk ratio *RR_RW_* of 4. (The subscript RW is used to denote the risk ratio of RSV illness associated with wheezing illness). In the placebo arm, applying these risks to the 200 and 800 infants with and without RSV illness, respectively, results in 80 total cases of recurrent wheezing among infants in the placebo arm ((200 × 0.20 + (800 × 0.05)). Among infants born to RSV-vaccinated mothers, 900 will have a 5% risk of developing the recurrent wheezing, and 100 will have a 20% risk, for a total of 65 cases ((900 × 0.05) + (100 × 0.2)). In this hypothetical scenario, maternal RSV immunization reduced the risk of childhood wheezing from 80 per 1000 (placebo arm) to 65 per 1000 (vaccine arm), for a risk ratio associated with vaccination (*RR_VW_*) of 0.81 (= 65 per 1000 ÷ 80 per 1000) (Fig. 2). (The subscript VW is used to denote the risk ratio of vaccine associated with wheezing illness, or the vaccine effect size). We used estimates from the literature supplemented by expert opinion to inform the following parameter inputs for our model: (1) baseline attack rate of severe early RSV, *r*; (2) vaccine efficacy, *VE*; (3) risk of recurrent wheezing during childhood, *w*; and (4) risk ratio for recurrent wheezing according to early RSV illness, *RR_RW_* (Fig. 1). We also considered two allocation schemes, 1:1 and 2:1.

### 2.2 Baseline attack rate of early severe RSV

Up to two-thirds of infants acquire RSV during their first year of life [5]. Only a proportion of these illnesses is preventable by maternal immunization, as passive protection from maternal antibodies wanes with time and are not expected to exceed 6 months [1]. For simplicity, we assumed that subclinical or mild early RSV illness would have a very weak association, if any, with development of later recurrent childhood wheezing. We found the most stable estimates of severe RSV incidence during this risk period were for infants younger than 6 months of age. Thus, we used estimates of the baseline RSV attack rate during the first 6 months of life that resulted in the more severe outcomes of LRI or hospitalization. In this report, we use “early severe RSV illness” to define RSV illness during the first 6 months of life that resulted in LRI or hospitalization.

A recent meta-analysis estimated that between 6.3% and 16.9% of infants acquired a RSV-related LRI in the first 6 months of life across world income strata [4]. The rate of hospitalization in countries with adequate access to medical care may best reflect the attack rate of the most severe illnesses. RSV-hospitalization estimates for most income strata were greater than 2% between 0 and 5 months of age, with the upper bound of 2.7%. We used values of 2.7%, 6.0%, and 17.0% as attack rates of early severe RSV illness.

### 2.3 Vaccine efficacy

There is no information currently available on the efficacy of candidate maternal RSV vaccines. The WHO Preferred Product Characteristics for RSV Vaccines specifies that a vaccine with 50% efficacy would be favourable, while greater than 70% efficacy would be preferred [1], [2]. We used values of 50.0%, 70.0%, and 90.0%.

### 2.4 Baseline risk of recurrent wheezing among children unexposed to early severe RSV illness

Estimates for recurrent childhood wheezing vary widely across countries and represent the overall rate of recurrent wheezing in a population, independent of early RSV status. We were unable to find global estimates of recurrent childhood wheeze for children aged up to 3 years. As a proxy, we used the International Study of Asthma and Allergies in Childhood (ISAAC) survey which reports the most recent international estimates for “symptoms of severe asthma” among children aged 6–7 years, which was the youngest age group ISAAC studied. Children had these symptoms if they had current wheeze and reported any of the following: ≥4 attacks of wheeze, ≥1 night/week of sleep disturbance from wheeze, or wheeze-affected speech, within the previous year. The global prevalence of these symptoms among this age group was 4.9%, with the highest prevalence in the Oceanic region (9.5%), and the highest centre rate in Costa Rica (20.3%). We used parameter inputs of 4.9%, 9.5%, and 20.0%.

We expect the actual prevalence of recurrent wheezing at 3 years to be higher than the prevalence of these symptoms reported among 6–7 years. Furthermore, these estimates do not account for early RSV exposure status, even though we require estimates among children unexposed to early severe RSV illness. These overall estimates will approximate the estimate among the unexposed when the exposed population is small (i.e., for small baseline attack rates of severe early RSV) or when RR_RW_ is low. Otherwise, they would be overestimates. We therefore chose a wide range of recurrent wheezing prevalence estimates to reflect these opposing measurement uncertainties.

### 2.5 Risk ratio of recurrent wheezing according to RSV status

If RSV vaccination during pregnancy leads to reduction in later recurrent childhood wheezing, we assume the effect occurs by preventing early severe RSV illness among infants. The gold standard study design for estimating a causal risk ratio between early severe RSV illness and recurrent childhood wheezing would be a RCT of RSV prevention with predefined outcomes, however there have been few such trials conducted [19], [20]. Several observational studies have assessed the risk of recurrent wheezing associated with RSV-related hospitalizations in infancy, but they are at risk of confounding bias, particularly from factors predisposing infants to severe RSV illness and later development of wheezing, such as poor lung function. A 2017 systematic review summarizing these studies reported risk ratios between 1.7 and 3.3 for recurrent wheezing occurring 3–5 years after RSV-hospitalization during infancy [21]. However, risk ratios as high as 4.3 were estimated for recurrent childhood wheezing after only one year of follow-up [21]. We used *RR_RW_* parameter inputs of 1.6, 2.6, and 4.0.

### 2.6 Calculation of effect size, sample size, and number needed to treat

For each combination of the parameter estimates (summarized in Table 1), we calculated the risk of recurrent childhood wheezing in each of the placebo and active vaccine arms. From these risks, we calculated *RR*_VW_, the corresponding risk ratio for maternal RSV vaccination and recurrent childhood wheezing. These risk ratios are the effect sizes that a trial would be designed to detect, with larger sample sizes needed to detect smaller effect sizes (i.e., *RR_VW_* closer to 1). We did not consider the time-varying relationships between gestational timing of maternal vaccination, birth, and RSV seasonality [22], as maternal vaccine RCTs with paediatric RSV endpoints are expected to time vaccination to maximize transplacental antibody transport and births occurring during the RSV season.

**Table 1:** Parameters used in sample size calculations.

Variable	Values	Rationale for chosen values
Severe early RSV attack rate	2.7%, 6.0%, 17.0%	While up to two-thirds of infants acquire RSV in their first year of life, many of these infections are subclinical. We assume that the RSV illnesses on the causal pathway to recurrent childhood wheezing are therefore a subset of the more severe illnesses. Shi et al [4] reported comprehensive, regional estimates for RSV-associated lower respiratory infections (RSV-LRI). These estimates span 63.3–168.9 RSV-LRI per 1000 infants (6.3–16.9%, rounded to 6.0% and 17.0% in our models) across the income regions. The lowest estimate (63.3/1000) was for lower-middle income countries, while the highest estimate was from upper-middle income countries (168.9/1000). However, the order of estimates was not aligned with income level (i.e., the lowest income countries had estimates between those of lower-middle income countries and upper-middle income countries). The lowest estimate was 0.7% from lower income countries, which we expect to be spuriously low due to due to decreased healthcare utilisation in such settings. Shi et al [4] also provided estimate for RSV-LRI hospitalizations. These estimates were >20.2/1000 for all income regions except the low-income region. 27.1/1000 (2.7%) reflects the estimate for industrialized regions. This may reflect the most appropriate attack rate if only the most severe RSV-LRI lead to later recurrent childhood wheezing

Vaccine efficacy	50.0%, 70.0%, 90.0%	There is no information on the potential efficacy on candidate RSV vaccines. However, the WHO specified that a vaccine with 50.0% efficacy would be considered, while greater than 70.0% efficacy is preferred [1]. We also consider a 90.0% efficacy point as an upper bound

Vaccine allocation scheme	1:1, 2:1	The allocation scheme denotes the number of mother-infant pairs randomized to receive vaccine vs. placebo. The chosen schemes (1:1 and 2:1) are the most common ones used in randomized controlled trials

Baseline risk of recurrent childhood wheezing	4.9%, 9.5%, 20.0%	Recurrent childhood wheezing prevalence varies substantially by country. The International Study of Asthma and Allergies in Childhood (ISAAC) reported prevalence rates of asthma, recurrent wheezing, and current wheezing for 6–7-year olds and 13–14-year olds [29]. The global rate of recurrent wheezing among 6–7-year olds is 4.9%. Regionally, the highest rate was 9.5% in Oceania, similar to the rate for other English-speaking centres. The highest centre rate was 20.3% in Costa Rica, while the lowest regional rate was 3.2% in Asia-Pacific. For the study we use 4.9%, 9.5%, and 20.0% to cover this range. Sample size requirements will be larger in areas with lower than a 4.9% baseline risk of recurrent wheezing

Risk ratio for the association between early RSV-LRI and recurrent childhood wheezing	1.6, 2.6, 4.0	A 2017 systematic review by Fauroux and colleagues examined the association between early RSV-LRI hospitalization (RSV-h) and asthma/wheezing in Western countries. The RSV-hospitalization occurred before 3 years of age, with most studies looking before 12 months, and asthma/wheezing was measured later, in the shortest studies wheezing was measured after only a year of life, while the longest studies measured asthma 30 years later. Most studies estimated a harmful association (risk ratios > 1). For studies with follow-up of 6 years or fewer, the provided or calculated risk ratios (RRs) ranged between 0.5 and 4.3. However, these were the RRs for RSV-LRI *hospitalizations*, suggesting that RRs for RSV-LRI *overall* would have been lower. Risk ratios for the studies reported in Fauroux, where the follow-up time is 6 years or less ranged from 0.5 to 4.3. Values used in the sample size calculations: 1.6, 2.6, 4.0

To calculate the sample size required to detect a difference between these risks, we performed a two-sided sample size calculation assuming 80% statistical power and 5% type I error, under a 1:1 or a 2:1 vaccine allocation to active vaccine and placebo arms, respectively. These calculations were performed using the *power.prop.test* function from the *stats* package, and the *bsamsize* function from the *Hmisc* package in R version 3.4.2 [23]. To estimate the number of pregnant women needed to be vaccinated to prevent one case of recurrent childhood wheezing, we calculated the absolute difference in risk of recurrent childhood wheezing in vaccinated and unvaccinated mother-infant pairs (i.e., the number of excess cases per 100 unvaccinated women) and took the inverse of this value.

### 2.7 Scenario plausibility

While the parameter inputs reflect a range of evidence-based estimates, some combinations of parameter values resulted in scenarios that were unlikely, particularly when multiple extreme parameters were considered simultaneously. Scenarios that combined a baseline risk of recurrent wheezing of 20.0% with a 4.0-fold increase in risk of recurrent wheezing following a severe early RSV illness were categorized as “least likely” since this would imply that 80% of children who had an early severe RSV illness would later develop recurrent wheezing, which we deemed unreasonably high. Furthermore, scenarios that combined a 2.6-fold or 4.0-fold increase in risk of recurrent wheezing and a 17.0% attack rate of early severe RSV illness were categorized as “less likely” or “least likely”, respectively. This is because these risk ratios estimates were based on the association between RSV-hospitalization and recurrent childhood wheezing, while 17.0% referred to all RSV-LRIs, not just hospitalizations (which is 6 times higher than the estimated RSV-hospitalization rate of 2.7%). All other scenarios were categorized as “more plausible”.

### 2.8 Data sharing

All of the statistical code to perform these calculations and reproduce this manuscript is publicly available on GitHub: https://github.com/corinne-riddell/RSV-wheeze-sample-size/.

### 2.9 Role of funding source

The funders played no role in study design, interpretation of the data, the writing of the report, or the decision to submit the manuscript for publication. The corresponding author had full access to all the data in the study and had final responsibility for the decision to submit for publication.

## 3 Results

There were 81 scenarios for each of the 1:1 and 2:1 allocation schemes. Under each scheme, 57 were categorized as more plausible, 9 as less likely, and 15 as least likely due to the presence of extreme parameters considered simultaneously (Table 2). Of all scenarios, 70% had vaccine effect sizes (*RR_VW_*) between 0.9 and 1.0, with effect sizes nearest to 1.0 being the most difficult to detect. The less likely and least likely scenarios had the lowest *RR_VW_* values.

**Table 2:** Estimated risk ratios for recurrent childhood wheezing in vaccinated vs. unvaccinated mother-infant pairs across several scenarios.^1^

		Risk ratio between RSV and recurrent childhood wheezing^2^		
Attack rate of severe early RSV^3^	Percent altered^4^	1.6	2.6	4.0
*Vaccine efficacy of 50%*				
2.7%	1.4%	0.99	0.98	0.96
6.0%	3.0%	0.98	0.96	0.92
17.0%	8.5%	0.95	0.89 (Less likely)	0.83 (Least likely)

*Vaccine efficacy of 70%*				
2.7%	1.9%	0.99	0.97	0.95
6.0%	4.2%	0.98	0.94	0.89
17.0%	11.9%	0.94	0.85 (Less likely)	0.76 (Least likely)

*Vaccine efficacy of 90%*				
2.7%	2.4%	0.99	0.96	0.93
6.0%	5.4%	0.97	0.92	0.86
17.0%	15.3%	0.92	0.81 (Less likely)	0.70 (Least likely)

*Notes*:

1 This table does not present recurrent wheezing, *w*, as a parameter input because it cancels out in the calculation of the risk ratios (implying that these risk ratios hold for every level of baseline risk of recurrent wheezing).

2 The risk ratios (RR_RW_) between RSV illness and recurrent childhood wheezing are estimated based on relationships between RSV-hospitalizations and later development of recurrent childhood wheezing. Scenarios that combined a baseline risk of recurrent wheezing of 20.0% with a 4.0-fold increase in risk of recurrent wheezing following a severe early RSV illness were categorized as “least likely”. Scenarios that combined a 4.0-fold or 2.6-fold increase in risk of recurrent wheezing and a 17.0% attack rate of early severe RSV illness were categorized as “least likely” or “less likely”, respectively. All other scenarios are considered “more plausible”.

3 The largest attack rates (6.0% and 17.0%) are estimated based on the proportion of infants that acquired RSV-LRI between 0 and 5 months from community-based studies, and the smallest attack rate corresponds to the portion of infants aged 0 to 5 months hospitalized for severe RSV.

4 The percent altered is the proportion of infants expected to have their RSV status affected by maternal vaccination during pregnancy in the vaccination arm and can be calculated by multiplying the vaccine efficacy by the attack rate of severe, early RSV illness.

**Fig. 3:**
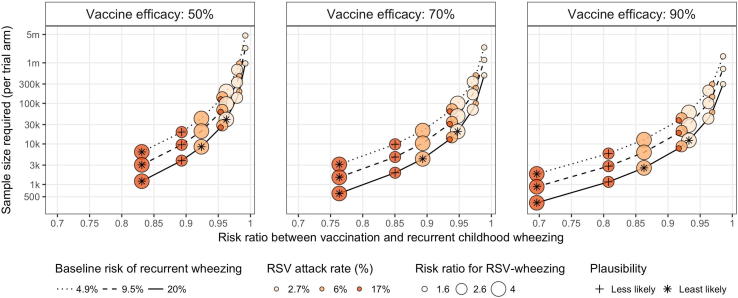
Minimal sample size required (per trial arm) to detect a difference in recurrent childhood wheezing for mother-infant pairs vaccinated against RSV under a 1:1 allocation scheme across several scenarios. This figure illustrates the estimated risk ratio between vaccination and recurrent wheezing (RR_VW_, on the x-axis) that results from the parameters that define each scenario, indicated by the size, colour, line type, and panel. The corresponding sample size to detect the risk ratio is shown on the y-axis, which is plotted on a log scale. Scenarios classified less likely are indicated with a cross (+), and those classified least likely are denoted with an asterisk (∗).

The sample size requirements increase as a function of vaccine effect size (*RR_VW_*)**,** becoming larger as the effect size diminishes. Fig. 3 illustrates the required sample size per trial arm for each scenario under the randomization scheme of 1:1. Among the more plausible scenarios, the sample size required per arm ranges between 6196 and 4.7 million, with 75% of plausible scenarios requiring more than 31,060 mother-infant pairs per trial arm, and 47% requiring more than 100,000 mother-infant pairs per trial arm. With a vaccine efficacy of 50%, the plausible scenarios would require at least 20,657 mother-infant pairs per arm to detect an effect of RSV maternal vaccination on recurrent wheezing. Less likely scenarios require between 1159 and 5799 pairs per arm among those with 90% vaccine efficacy, and between 3878 and 19,623 pairs per arm among those with 50% vaccine efficacy. The least likely scenarios require sample sizes ranging between 354 and 39,990 per trial arm. The total sample size under each scenario is provided in Table 3. Larger sample sizes would be required for study designs using 2:1 randomization as compared to 1:1 randomization (Table 3).

**Table 3:** Estimated risk ratios and total sample size requirements for each scenario according to their randomization scheme.^1^





*Note*:

^1^Scenarios that combined a baseline risk of recurrent wheezing of 20.0% with a 4.0-fold increase in risk of recurrent wheezing following a severe early RSV illness were categorized as “least likely”. Scenarios that combined a 4.0-fold or 2.6-fold increase in risk of recurrent wheezing and a 17.0% attack rate of early severe RSV illness were categorized as “least likely” or “less likely”, respectively. “Least likely” scenarios are shaded in dark grey and “less likely” scenarios are shaded in light grey.

^2^In the report text, these abbreviations for the parameters were used: w (baseline risk of recurrent wheezing among unexposed at 3 years), RR_RW_ (risk ratio for RSV- recurrent childhood wheezing), RR_VW_ (risk ratio for vaccination- recurrent childhood wheezing, or the vaccine effect size).

**Fig. 4:**
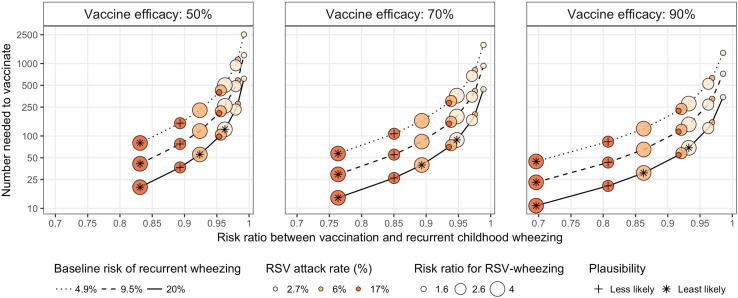
Number needed to vaccinate to prevent one case of recurrent childhood wheezing under a 1:1 allocation scheme across several scenarios. This figure illustrates the estimated risk ratio between vaccination and recurrent wheezing (RR_VW_, on the x-axis) that results from the parameters that define each scenario, as indicated by the size, colour, line type, and panel. The corresponding number of pregnant women requiring vaccination to prevent one case of recurrent childhood wheezing is shown on the y-axis, which is plotted on a log scale. Scenarios classified less likely are indicated with a cross (+), and those classified least likely are denoted with an asterisk (∗).

Fig. 4 displays the number of women who need to be vaccinated in order to prevent one case of recurrent childhood wheezing under each of the considered scenarios with 1:1 vaccine allocation. Among the more plausible scenarios, at least 54 women need to be vaccinated to prevent one case. Seventy-five percent of these plausible scenarios require at least 147 women to prevent one case, while 25% require more than 503 women. Scenarios classified as less likely require between 20 and 150 women, while scenarios classified as least likely require between 10 and 123 women. The number needed to vaccinate for each allocation scenario is provided in Table 3.

## 4 Discussion

Most maternal RSV immunization scenarios that we evaluated resulted in very small vaccine effect sizes measuring the impact on recurrent childhood wheezing (i.e., RR_VW_ > 0.9) in general populations, and they would require unfeasibly large sample sizes to detect significant differences between trial arms. The scenarios considered more plausible required sample sizes of at least 6196 per trial arm, with most of these scenarios requiring substantially larger sample sizes. All of the more plausible scenarios exceed the size of the only current phase III trial of RSV vaccination in pregnant women, which has a total planned sample size of 8618 randomized in 2:1 vaccine to placebo allocation [24]. Our sample size calculations indicate the improbability of RCTs conducted for licensure of RSV vaccines for use by pregnant women in general populations to prevent recurrent childhood wheezing. Trials with asthma endpoints would likely need to be even larger.

While our goal was to inform public health about the potential impact of RSV vaccines during pregnancy, our findings may be generalized to trials of anti-RSV monoclonal antibodies or paediatric RSV vaccination. Two RSV monoclonal antibody RCTs designed to demonstrate prevention of RSV illness in infants were extended to monitor participants for medically-attended wheezing at 3 years [19] or asthma at 6 years [20]. Both trials were powered to detect RSV prevention on a reduction in wheezing days within the first year of life, and not later childhood respiratory outcomes. In a RCT conducted among 2127 Native American infants in the United States, 14.9% of children in the treated group and 14.0% in the placebo group experienced serious early childhood wheezing between 1 and 3 years, of which only 3% in either arm had 3 or more medically-attended wheezing episodes [25]. In the RCT conducted in the Netherlands, of 429 children who were followed to age 6, 10.3% in the RSV prevention arm had physician-diagnosed asthma at 6 years vs. 9.9% in the placebo arm. No difference in mean forced expiratory volume was detected [20]. Our results indicate that these studies were substantially smaller than what would be necessary to demonstrate a significant effect on RSV-caused respiratory outcomes. Furthermore, the small differences in respiratory outcomes across treatment arms suggest that even larger sample sizes would be required if the true RR_RW_ is lower than 1.6, the lower bound of the parameter included in this study.

Whether early RSV illness causes later recurrent wheezing is still unclear. Even assuming a moderate to strong risk ratio of RSV illness associated with wheezing illness (RR_RW_ between 1.6 and 4.0), our calculations indicate that the magnitude of vaccine effect on recurrent wheezing is likely small. However, the global burden of asthma as a lifelong chronic disease is sufficiently large that even a small reduction in asthma incidence would have major public health implications. Alternative study designs can overcome the sample size constraints of RCTs, and have been used to estimate the causal effect of maternal vaccination on a spectrum of childhood outcomes. However, caution should be taken in the design and interpretation of observational vaccine effectiveness studies. For example, confounding by health status led to unrealistic effect estimates of influenza vaccination on pneumonia among the elderly [26], and such studies still influence current global vaccine policy recommendations [27]. More objective measures of wheezing and asthma outcomes, such as physician’s diagnosis and lung function studies, can minimize differential measurement bias that can occur with parental reporting [28]. Planning in advance by engaging clinical experts, vaccinologists, and observational research methodologists is crucial to increase the quality and reliability of studies to best inform public health decisions.

Our investigation has its limitations. This analysis did not account for loss to follow-up, which is likely to occur for multiyear studies. For the purposes of our analyses, we assumed a causal link between infant RSV exposure and childhood recurrent wheezing, and the parameter choices represented a range of published estimates. Although we used estimates from systematic reviews, meta-analyses, and ISAAC surveys to obtain parameter inputs for our model, each of these is subject to its own weaknesses. While most stable estimates of severe RSV incidence were only available for infants younger than 6 months of age, passive protection from maternal antibodies wanes with time and may only last up to 2 months of age [13]. Data from the ongoing phase III clinical trial of RSV vaccination in pregnant women will provide valuable information on severe, early RSV attack rate and on vaccine efficacy which may refine our estimates once they are published [24]. To evaluate the risk of recurrent wheezing, we used prevalence estimates for symptoms of severe asthma (which includes recurrent wheezing) for 6–7 year-olds, which likely underestimate the prevalence among 3 year-olds. However, we calculated sample sizes for a wide range of inputs for recurrent wheezing to underscore its uncertainty and worldwide variation. Practically speaking, an even earlier recurrent wheezing endpoint (i.e., at 12–24 months of age) may be desired to keep trial costs and participant attrition low. However, diagnosis of wheezing is subjective and variable at these ages, and we were unable to find suitable disease rate estimates to investigate these earlier endpoints. If studies are planned to consider recurrent wheeze outcomes in children 2–3 years of age, and baseline rates are known, our tables may still provide guidance about whether sample size is sufficient so long as the expected parameter values are close to those we investigated.

A safe maternal RSV vaccine that prevents acute RSV illness in early infancy would be a major public health accomplishment due to the high burden of RSV illness in young children globally. Based on our analysis, RCTs for candidate maternal RSV vaccines undertaken for licensure are unlikely to demonstrate an effect on recurrent wheezing illness due to the large sample sizes needed to demonstrate a significant effect. Further efforts are needed to plan for post-licensure studies to inform public health expectations regarding the impact of RSV vaccines given during pregnancy.

## 5 Contributors

JRO conceived of the study. CAR and JRO designed the study. CAR, NB, LJB, WDD, DRF, DBF, TG, TVH, JAH, RAK, HN, RCR, TS, PDS, RTS, PW, HJZ, and JRO reviewed model inputs. CAR performed the statistical analyses. CAR and JRO drafted the manuscript. CAR, NB, LJB, WDD, DRF, DBF, TG, TVH, JAH, RAK, HN, RCR, TS, PDS, RTS, PW, HJZ, and JRO critically revised the manuscript. All authors had full access to study data, opportunity to review drafts, and approved the final version submitted for publication.

## References

[1] World Health Organization. Preferred Product Characteristics for Respiratory Syncytial Virus (RSV) Vaccines. Geneva, Switzerland: World Health Organization; 2017.

[2] Vekemans J., Moorthy V., Giersing B., Friede M., Hombach J., Arora N. (2018). Respiratory syncytial virus vaccine research and development: World Health Organization technological roadmap and preferred product characteristics. Vaccine.

[3] Nunes M.C., Cutland C.L., Jones S., Downs S., Weinberg A., Ortiz J.R. (2017). Efficacy of maternal influenza vaccination against all-cause lower respiratory tract infection hospitalizations in young infants: results from a randomized controlled trial. Clin Infect Dis.

[4] Shi T., McAllister D.A., O'Brien K.L., Simoes E.A.F., Madhi S.A., Gessner B.D. (2017). Global, regional, and national disease burden estimates of acute lower respiratory infections due to respiratory syncytial virus in young children in 2015: a systematic review and modelling study. Lancet (London, England).

[5] Glezen W.P., Taber L.H., Frank A.L., Kasel J.A. (1986). Risk of primary infection and reinfection with respiratory syncytial virus. Am J Dis Child.

[6] Hall C.B., Weinberg G.A., Iwane M.K., Blumkin A.K., Edwards K.M., Staat M.A. (2009). The burden of respiratory syncytial virus infection in young children. New England J Med.

[7] Caballero M.T., Jones M.H., Karron R.A., Hartert T.V., Simoes E.A., Stein R.T. (2016). The impact of respiratory syncytial virus disease prevention on pediatric asthma. Pediatr Infect Dis J.

[8] Karron R.A., Zar H.J. (2017). Determining the outcomes of interventions to prevent respiratory syncytial virus disease in children: what to measure?. Lancet Respir Med.

[9] Pavord I.D., Beasley R., Agusti A., Anderson G.P., Bel E., Brusselle G. (2018). After asthma: redefining airways diseases. Lancet.

[10] Global Initiative for Asthma. Global Strategy for Asthma Management and Prevention, 2018; 2018.

[11] Blanken M.O., Rovers M.M., Molenaar J.M., Winkler-Seinstra P.L., Meijer A., Kimpen J.L. (2016). Respiratory syncytial virus and recurrent wheeze in healthy preterm infants. New England J. Med.

[12] Martinez F.D. (2002). Development of wheezing disorders and asthma in preschool children. Pediatrics.

[13] Nunes M.C., Cutland C.L., Jones S., Hugo A., Madimabe R., Simoes E.A. (2016). Duration of infant protection against influenza illness conferred by maternal immunization: secondary analysis of a randomized clinical trial. JAMA Pediatr.

[14] Wright A.L. (2002). Epidemiology of asthma and recurrent wheeze in childhood. Clin Rev Allergy Immunol.

[15] Alvarez-Alvarez I., Niu H., Guillen-Grima F., Aguinaga-Ontoso I. (2018). Meta-analysis of prevalence of wheezing and recurrent wheezing in infants. Allergol Immunopathol.

[16] Kusel M.M., Kebadze T., Johnston S.L., Holt P.G., Sly P.D. (2012). Febrile respiratory illnesses in infancy and atopy are risk factors for persistent asthma and wheeze. Eur Resp J.

[17] Sly P.D., Boner A.L., Bjorksten B., Bush A., Custovic A., Eigenmann P.A. (2008). Early identification of atopy in the prediction of persistent asthma in children. Lancet.

[18] Hutcheon J.A., Fell D.B., Jackson M.L., Kramer M.S., Ortiz J.R., Savitz D.A. (2016). Detectable risks in studies of the fetal benefits of maternal influenza vaccination. Am J Epidemiol.

[19] O'Brien K.L., Chandran A., Weatherholtz R., Jafri H.S., Griffin M.P., Bellamy T. (2015). Efficacy of motavizumab for the prevention of respiratory syncytial virus disease in healthy Native American infants: a phase 3 randomised double-blind placebo-controlled trial. Lancet Infect Dis.

[20] Scheltema N.M., Nibbelke E.E., Pouw J., Blanken M.O., Rovers M.M., Naaktgeboren C.A. (2018). Respiratory syncytial virus prevention and asthma in healthy preterm infants: a randomised controlled trial. Lancet Resp Med.

[21] Fauroux B., Simoes E.A.F., Checchia P.A., Paes B., Figueras-Aloy J., Manzoni P. (2017). The burden and long-term respiratory morbidity associated with respiratory syncytial virus infection in early childhood. Infect Dis Ther.

[22] Savitz D.A., Fell D.B., Ortiz J.R., Bhat N. (2015). Does influenza vaccination improve pregnancy outcome? Methodological issues and research needs. Vaccine.

[23] R Development Core Team. R: A language and environment for statistical computing. R Foundation for Statistical Computing; 2017.

[24] ClinicalTrials.gov. A study to determine the safety and efficacy of the RSV F vaccine to protect infants via maternal immunization. In: Medicine NLo, editor. US.

[25] ClinicalTrials.gov. MEDI-524 (Motavizumab) for the Prevention of Respiratory Syncytial Virus (RSV) Disease Among Native American Indian Infants in the Southwestern United States. July 21, 2005; 2012.

[26] Jackson M.L., Nelson J.C., Weiss N.S., Neuzil K.M., Barlow W., Jackson L.A. (2008). Influenza vaccination and risk of community-acquired pneumonia in immunocompetent elderly people: a population-based, nested case-control study. Lancet (London, England)..

[27] Fifty-Sixth World Health Assembly. Prevention and control of influenza pandemics and annual epidemics; 2003.

[28] Scheltema N.M., Gentile A., Lucion F., Nokes D.J., Munywoki P.K., Madhi S.A. (2017). Global respiratory syncytial virus-associated mortality in young children (RSV GOLD): a retrospective case series. Lancet Glob Health.

[29] Lai C.K., Beasley R., Crane J., Foliaki S., Shah J., Weiland S. (2009). Global variation in the prevalence and severity of asthma symptoms: phase three of the International Study of Asthma and Allergies in Childhood (ISAAC). Thorax.

